# Measuring screen time among adolescents: test–retest reliability of HBSC questionnaire items across two countries

**DOI:** 10.1186/s12889-025-25950-9

**Published:** 2025-12-18

**Authors:** Michaela Matusova, Marek Maracek, Jan Pavelka, Kwok Ng, Catalina Medina, Nikola Tylsarova, Jens Bucksch, Zdenek Hamrik

**Affiliations:** 1 Faculty of Physical Culture, Palacky University Olomouc, Olomouc, Czech Republic; 2 Centre of Sports Activities, Brno University of Technology, Brno, Czech Republic; 3 Physical Activity for Health Research Centre, Department of Physical Education and Sport Sciences, Health Research Institute, University of Limerick, Limerick, Ireland; 4 Faculty of Education, University of Turku, Turku, Finland; 5 Institute of Sports Science and Innovation, Lithuanian Sports University, Kaunas, Lithuania; 6 National Institute of Public Health, Cuernavaca, Morelos, Mexico; 7 Department of Prevention and Health Promotion, Faculty of Natural and Social Sciences, Heidelberg University of Education, Heidelberg, Germany

**Keywords:** Children, Screen time, HBSC, Psychometrics, Questionnaire reliability

## Abstract

**Background:**

Increasing recreational screen time among adolescents is linked to adverse health outcomes like obesity and poor mental health. This highlights the need for reliable tools to monitor screen-based behaviours. The present study examined the test–retest reliability of recreational screen-time items from the Health Behaviour in School-aged Children (HBSC) questionnaire across culturally diverse adolescent populations.

**Methods:**

Using a test–retest design with a 2–3 week interval, we collected data from 750 adolescents (48.8% boys, mean age 15.29 years, SD 2.37) in Mexico (*n* = 233, aged 10–15y) and Czechia (*n* = 517, aged 10–18y) in 2022–2024. Self-reported time spent on gaming, social networking, video watching, and internet browsing were evaluated using Intraclass Correlation Coefficients (ICCs) for continuous measures and Cohen’s kappa for dichotomized outcomes (< 2 vs. ≥ 2 h/day), with analyses stratified by age, gender, and country.

**Results:**

Gaming and social networking demonstrated moderate-to-good reliability (ICC = 0.70–0.74, κ = 0.64–0.65, 82–83% unchanged responses). Video watching and browsing were less stable (ICC = 0.52–0.63, κ = 0.41–0.47). Czech primary school students exhibited the highest consistency (ICC = 0.76–0.81), while Mexican students completed the items with lower reliability (ICC = 0.43–0.54). Older adolescents (16–18 years) and girls reported greater stability for gaming and social networking, respectively.

**Conclusions:**

The screen-time items tested in this study showed acceptable test–retest reliability across countries, age groups, and sexes, particularly for gaming and social networking. These findings support their use in global adolescent health surveillance, while highlighting the need for refinement of less stable domains such as video watching and internet browsing. Given that samples were not nationally representative, findings should be interpreted within these specific contexts. Future research should enhance measurement precision and inform public health efforts to monitor and address screen-time related health risks.

## Background

Over the past decades, children’s screen time has increased substantially globally [1]. Compared to previous generations, today’s children spend significantly more time engaged in screen-based activities [2], with recreational screen time—unrelated to school or work—constituting a particularly prominent part of daily life [3]. Screen-based activities have become a dominant form of leisure among children and adolescents in contemporary societies. Digital screen use displaces a wide spectrum of non-digital activities, ranging from outdoor play and in-person social interactions to offline leisure activities like reading or creative play [4]. In the United States, for instance, children aged 8 to 18 years reportedly spend an average of 7.5 h per day watching or using screens [5]. International trends similarly point to a marked increase in adolescents’ recreational screen time and this pattern was observed across many different countries [1, 6]. Regional studies also show distinct socio-economic patterns; for example, research from Mexico and other Latin American countries indicates that higher-income adolescents often report more screen use and lower physical activity, contrasting trends seen in high-income countries [7].

Recreational and passive screen use (etc. video watching, gaming, scrolling through TikTok or Instagram), has been consistently associated with more negative outcomes in terms of sleep quality, mental health, and overall well-being, compared to educational or creative screen activities (e.g., homework, writing, content creation) [8–10]. Evidence indicates that engaging in more than two hours of recreational screen time per day is associated with adverse outcomes, including higher odds of obesity [3, 11], cardiometabolic risk [12, 13], poorer mental health [14, 15], disrupted sleep [16], and unhealthy eating habits [17]. Prolonged screen use has also been linked to anxiety, depression, impaired social skills [18, 19], and the displacement of health-promoting activities such as play, physical activity, homework, or sleep [20]. Importantly, different forms of screen time may carry distinct risks: for example, television viewing has been associated with headaches and irritability, while intensive gaming is more often linked to musculoskeletal issues and sleep problems [21].

Together, the findings from earlier research underline the importance to systematically monitor screen time activities among children and adolescents. Accurately measuring screen time is essential for public health surveillance, behavioural research, and the development of targeted interventions. Such data provide a foundation for identifying temporal trends, high-risk groups, and evaluating the outcomes of health promoting activities [18, 22].

In recent years, various sedentary behaviour questionnaires have been developed to assess screen time in different age groups and populations [23–28] . Systematic reviews show that although many of these instruments demonstrate acceptable test–retest reliability, evidence on their validity is often limited or inconsistently reported [29, 30]. Recent work further underlines the need for practical, validated tools that capture not only the duration but also the content and context of screen media use across diverse populations [31, 32].

Data on screen time are also available from large international studies such as the Health Behaviour in School-aged Children (HBSC) [33] and Global School-based Student Health Survey (GSHS) [34]. Although the primary focus of these studies is not screen time and there is a high need for short and simple research tools, these studies provide valuable insights into the trends of adolescents’ screen use behaviour. The structure of screen time questions in the HBSC has evolved over time to better capture adolescents’ changing digital habits. Initially, the focus was on TV viewing and computer use, with separate items for weekdays and weekends. From 2006 to 2014, the questionnaire expanded to include distinct items for watching TV, gaming, and other computer use, with a wide range of response options [1, 35] the questionnaire was further amended in 2018 and 2022 survey rounds [36].

Several studies have examined the test–retest reliability of screen time measures similar to those used in HBSC. For example, Kohoutek et al. [37] found moderate to good reliability among Vietnamese adolescents (ICCs = 0.51–0.72,κ = 0.42–0.53), and Liu et al. [38] reported satisfactory stability over three weeks in a Chinese sample. In Mexico, an earlier validation study demonstrated acceptable reliability of screen-time questions [39], but the items reflected technologies that are now largely outdated (e.g., Atari, GameBoy, email use). This further underscores the need to validate updated screen-time items and to examine the performance of the most widely used international surveillance items in contemporary, diverse adolescent populations.

Therefore, the main aim of the present study was to assess the test–retest reliability of screen time measures currently used in the HBSC questionnaire. Specifically, the study sought to examine the consistency of adolescents’ responses to recreational screen time-related items over an 2–3 weeks-interval, different demographic groups and cultures. By addressing this gap, the study contributes to ongoing efforts to improve the quality of screen time surveillance and supports the development of more robust, reliable tools for international health monitoring among adolescents.

## Sample and procedures

The study employed a test–retest design with an interval of 2–3 weeks between the initial test and retest to assess reliability over this period. Data collection was through online surveys and occurred across distinct cohorts and regions. In Mexico, testing spanned from April to June 2022, targeting primary and secondary private school students (10–15 years). In Czechia, data were gathered from high schools (16–18 years) in May to June 2022 and from primary schools (10–16 years) in June 2024.

In Mexico, the research was conducted in private schools located in an urban area in the south of Mexico City. Before data collection, the main objectives and procedures of the study were explained to the parents. The children and their parents signed the assent and consent forms before data collection began. Questionnaires were administered by trained personnel during school hours. For data collection, the schools provided a classroom and a computer, and the process was conducted without supervision from teachers or parents. A total of 310 students were eligible to participate, with 236 completing both the test and retest, yielding a completion rate of 76.1%. After excluding respondents with missing data, the final dataset consists of 210 students.

In Czechia, six public high schools were randomly selected from the database of high schools in the Olomouc Region, half of the schools were located in the city of Olomouc (≈100,000 inhabitants), while the remaining schools were situated in smaller towns of approximately 10,000 inhabitants. Questionnaires were administered by trained research assistants during regular school hours to students in the 2nd and 3rd grades (488 registered students) and without teacher supervision to minimize bias. The initial test phase yielded responses from 417 adolescents (response rate: 85.45%). The retest phase included 371 respondents (response rate: 76.02%). Due to the non-completion of the second questionnaire and inability to match participant IDs, 91 of participants were excluded. After removing cases with missing data and participants older than 18.99 years, the final sample consisted of 307 Czech adolescents (47.2% girls).

For the primary school cohort in Czechia (June 2024), data were collected from 266 students from Olomouc and Usti and Labem regions (50% rural schools; response rate: 84.95%, 319 students registered in classes) in the test and 271 in the retest (response rate: 84.01%). Paired test–retest sample include 221 adolescents. The final sample include 210 adolescents.

### Measures

The study assessed recreational screen-time behaviours through a self-report questionnaire, using items from the Health Behaviour in School-aged Children (HBSC) 2021/22 survey protocol [33]. Participants were asked to estimate daily hours spent on the following: (1) playing games on devices such as computers, game consoles, tablets, smartphones, or smart TVs; (2) using computers or electronic devices for social networking (e.g., Instagram, Facebook, Twitter, Snapchat); (3) watching TV, DVDs, or videos, including online platforms like YouTube; and (4) browsing the internet or seeking information online. Response options ranged from: is “None at all,” “About half an hour a day,” “About 1 h a day,” “About 2 h a day,” “About 3 h a day,” “About 4 h a day,” “About 5 h a day,” “About 6 h a day,” to “About 7 or more hours a day.” These were recorded into continuous values (0, 0.5, 1, 2, 3, 4, 5, 6, 7 h) to reflect approximate hours spent daily. After recording, a cumulative recreational screen-time variable was created by summing the continuous values for gaming, social networking, watching videos, and browsing the internet. For categorical analyses, a cut-off of 2 h per day was applied to each domain of recreational screen time, following earlier HBSC studies [1, 35]. Adolescents reporting less than 2 h per day were classified as meeting the recommended threshold, and those reporting 2 h or more as not meeting it. For the composite indicator across all four domains, a threshold of 4 h per day was used, with participants categorized as either < 4 h/day or ≥ 4 h/day.

### Statistical analysis

Data analyses were conducted using IBM SPSS (version 28) and Python (version 3.12.3). To assess the test–retest reliability of adolescents’ self-reported screen-time behaviours, both continuous and dichotomous approaches were employed. Intraclass Correlation Coefficients (ICCs) were calculated for continuous variables (e.g., average daily hours) using a two-way mixed-effects model with absolute agreement for single measurements. Given that the screen-time items were originally measured on an ordinal response scale, the analyses treated these variables as continuous for the ICC computation, assuming interval-level measurement; this assumption should be considered when interpreting ICC values. Based on commonly accepted thresholds, ICC values below 0.5 indicate poor reliability, values between 0.5 and 0.75 indicate moderate reliability, values between 0.75 and 0.9 indicate good reliability, and values above 0.90 indicate excellent reliability [40]. For dichotomized outcomes (screen time < 2 h vs. ≥ 2 h per day,for cumulative variable < 4 h and ≥ 4 h per day), Cohen’s kappa coefficients (κ) were calculated to assess agreement beyond chance. Additionally, the proportion of participants who remained in the same category across both time points (% no shift) was reported to provide a complementary index of response stability. As outlined by Cohen [41], Cohen’s Kappa values are interpreted as follows: values above 0.5 reflect a large level of agreement, values between 0.3 and 0.5 indicate moderate agreement, values from 0.1 to 0.3 suggest small agreement, and values below 0.1 are considered trivial.

Reliability (ICC for continuous; Kappa for dichotomized variables) was examined for four types of screen-based activity: gaming, social networking, watching TV/videos, browsing the internet and a composite sum score. Analyses were conducted on the combined dataset as well as separately for each data collection: Czech primary schools, Mexican private primary and secondary schools, and Czech high schools. Test–retest consistency was further explored across gender (boys vs. girls) and age groups (10–12, 13–15, and 16–18 years), allowing for subgroup comparisons of reliability indicators.

## Results

The final sample (Table 1) consisted of 750 adolescents (48.8% boys), with a mean age of 15.29 years (SD = 2.37). The Czech sample included two subgroups. The first subgroup consisted of 307 adolescents attending grammar school, aged 16–18 years (M = 17.77, SD = 0.58, 52.8% boys). The second subgroup comprised 210 adolescents from primary schools, aged 10–16 years (M = 13.95, SD = 1.51, 50.0% boys). The Mexican sample consisted of 233 participants aged 10–15 years (M = 13.23, SD = 1.28, 42.5% boys).

**Table 1:** Descriptive characteristics of sample

	**Boy**	**Girl**	**10-12y**	**13-15y**	**16-18y**	**M (SD)**
CZ primary school (*N* = 210)	105 (50.0%)	105 (50.0%)	60 (28.6%)	141 (67.1%)	9 (4.3%)	13.95 (1.51)
CZ high school (*N* = 307)	162 (52.8%)	145 (47.2%)	0 (0.0%)	0 (0.0%)	307 (100.0%)	17.77 (0.58)
MX primary and secondary school (*N* = 233)	99 (42.5%)	134 (57.5%)	99 (42.5%)	133 (57.1%)	1 (0.4%)	13.23 (1.28)
Total (*N* = 750)	366 (48.8%)	384 (51.2%)	159 (21.2%)	274 (36.5%)	317 (42.3%)	15.29 (2.37)

*CZ *Czechia, *MX* Mexico

Table 2 presents the proportion of adolescents who reported engaging in less than two hours per day of various screen-based activities, at both the test and retest assessments. Across the full sample, 78.8% and 82.3% of adolescents reported less than two hours per day of internet browsing at test and retest, respectively. For watching TV/videos, gaming, and social networking, the proportions were 52.0% and 56.5%, 55.5% and 54.5%, and 41.2% and 42.8%, respectively.

**Table 2:** Proportion of adolescents (%) reporting < 2 h/day of screen time in each activity domain, and < 4 h/day for cumulative screen time

	**Gaming** (< 2 h/day)		**Social networking** (< 2 h/day)		**Watching video** (< 2 h/day)		**Browsing internet** (< 2 h/day)		**Cumulative time** (< 4 h/day)	
	**Test**	**Retest**	**Test**	**Retest**	**Test**	**Retest**	**Test**	**Retest**	**Test**	**Retest**
	n (%)	n (%)	n (%)	n (%)	n (%)	n (%)	n (%)	n (%)	n (%)	n (%)
CZ primary school	88 (41.9%)	92 (43.8%)	74 (35.2%)	84 (40.0%)	89 (42.4%)	93 (44.3%)	177 (84.3%)	191 (91.0%)	23 (11.0%)	32 (15.2%)
CZ high school	200 (65.2%)	196 (63.8%)	126 (41.0%)	128 (41.7%)	167 (54.4%)	190 (61.9%)	246 (80.1%)	253 (82.4%)	83 (27.0%)	90 (29.3%)
MX primary and secondary school	128 (54.9%)	121 (51.9%)	109 (46.8%)	109 (46.8%)	134 (57.5%)	141 (60.5%)	168 (72.1%)	173 (74.3%)	54 (23.2%)	58 (24.9%)
Total	416 (55.5%)	409 (54.5%)	309 (41.2%)	321 (42.8%)	390 (52.0%)	424 (56.5%)	591 (78.8%)	617 (82.3%)	160 (21.3%)	180 (24.0%)

*CZ* Czechia, *MX* Mexico

Differences between subgroups were observed across educational settings. Among Czech primary school students, 84.3% at test and 91.0% at retest reported less than two hours per day of internet browsing. For watching TV/videos, gaming, and social networking, the proportions spending less than two hours per day were lower in this subgroup than in the other groups. Among Czech high school students, 65.2% at test and 63.8% at retest reported less than two hours per day of computer gaming. In the Mexican sample, 46.8% of students reported using social networking sites, and a higher proportion reported spending more than two hours per day browsing the internet than in the Czech subgroups.

**Fig. 1:**
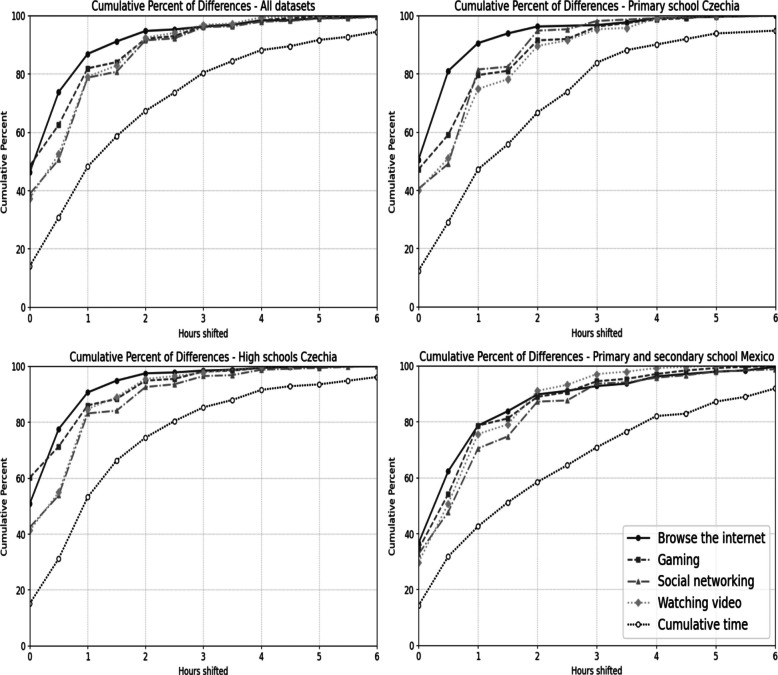
Cumulative graph of shift between test and retest 0–6 h

Figure 1 presents a cumulative graph illustrating the magnitude of change between the test and retest. The patterns observed are similar between Czechia and Mexico. For individual variables, no change was observed in approximately 30–60% of participants. In all cases, when considering a difference of 0–1 h, the proportion of respondents approaches or, in some cases, exceeds 80%. For the cumulative screen time variable, exact test–retest agreement was observed in approximately 15% of participants. When allowing for a difference of 0–2 h, the proportion increases to at least 60%.

There was moderate test–retest reliability for overall screen time, with intraclass correlation coefficients (ICCs) typically ranging from 0.50 to 0.75. Cohen’s kappa values interpreted as moderate to large agreement. The ICC (Table 3) for gaming time was 0.74 (95% CI [0.71, 0.77]), interpreted as moderate reliability, and the corresponding kappa coefficient (Table 4) was 0.65. Furthermore, 82.5% of participants reporting the same category at both time points. The social networking item also had moderate reliability (ICC = 0.70, 95% CI [0.66, 0.73]) and a high level of categorical agreement (κ = 0.64), with 82.67% of participants remaining in the same category. This pattern is held across most subgroups, with moderate to good reliability and substantial agreement. Cumulative screen time also demonstrated moderate reliability (ICC = 0.74, 95% CI [0.71, 0.77]) and moderate categorical agreement (κ = 0.58), with 85.33% of participants reporting the same category at both time points.

**Table 3:** Test–retest reliability of screen time behaviours measured using ICC and 95%-Confidence Interval (CI)

		**All**			**Boy**			**Girl**			**10–12**			**13–15**			**16–18**		
		**ICC**	**LCI**	**UCI**	**ICC**	**LCI**	**UCI**	**ICC**	**LCI**	**UCI**	**ICC**	**LCI**	**UCI**	**ICC**	**LCI**	**UCI**	**ICC**	**LCI**	**UCI**
All datasets	Gaming	0.74	0.71	0.77	0.75	0.70	0.79	0.65	0.59	0.71	0.73	0.65	0.79	0.66	0.58	0.72	0.80	0.76	0.84
	Social networking	0.70	0.66	0.73	0.65	0.59	0.71	0.72	0.67	0.77	0.74	0.66	0.80	0.66	0.58	0.72	0.67	0.61	0.73
	Watching video	0.63	0.59	0.67	0.60	0.53	0.66	0.66	0.60	0.71	0.69	0.60	0.76	0.56	0.47	0.64	0.67	0.61	0.73
	Browsing internet	0.52	0.46	0.57	0.55	0.48	0.62	0.47	0.39	0.55	0.55	0.44	0.65	0.44	0.34	0.53	0.60	0.53	0.67
	Cumulative time	0.74	0.71	0.77	0.73	0.68	0.78	0.74	0.69	0.78	0.81	0.74	0.86	0.68	0.61	0.74	0.74	0.68	0.79
CZ primary school	Gaming	0.76	0.70	0.81	0.79	0.71	0.85	0.67	0.55	0.77	0.80	0.68	0.87	0.74	0.66	0.81	0.60^a^	−0.04^a^	0.89^a^
	Social networking	0.81	0.75	0.85	0.79	0.71	0.86	0.80	0.72	0.86	0.81	0.70	0.88	0.78	0.70	0.83	0.76^a^	0.25^a^	0.94^a^
	Watching video	0.65	0.56	0.72	0.69	0.57	0.78	0.61	0.47	0.72	0.73	0.58	0.83	0.61	0.49	0.70	0.78^a^	0.29^a^	0.95^a^
	Browsing internet	0.53	0.42	0.62	0.51	0.36	0.64	0.55	0.40	0.67	0.66	0.49	0.78	0.42	0.28	0.55	*0.70*^a^	*0.12*^a^	*0.92* ^a^
	Cumulative time	0.81	0.76	0.85	0.82	0.75	0.88	0.80	0.72	0.86	0.88	0.81	0.93	0.78	0.70	0.83	*0.68*^a^	*0.09*^a^	*0.92*^a^
CZ high school	Gaming	0.81	0.76	0.84	0.77	0.69	0.82	0.68	0.58	0.76							0.81	0.76	0.84
	Social networking	0.67	0.60	0.73	0.61	0.51	0.70	0.75	0.67	0.81							0.67	0.60	0.73
	Watching video	0.66	0.59	0.72	0.56	0.45	0.66	0.77	0.70	0.83							0.66	0.59	0.72
	Browsing internet	0.60	0.52	0.67	0.61	0.51	0.70	0.57	0.44	0.67							0.60	0.52	0.67
	Cumulative time	0.73	0.68	0.78	0.70	0.61	0.77	0.74	0.65	0.80							0.73	0.68	0.78
MX primary and secondary school	Gaming	0.59	0.50	0.67	0.64	0.51	0.74	0.52	0.38	0.63	0.65	0.53	0.75	0.54	0.41	0.65			
	Social networking	0.63	0.54	0.70	0.52	0.36	0.65	0.64	0.52	0.73	0.71	0.60	0.80	0.52	0.39	0.63			
	Watching video	0.54	0.44	0.62	0.48	0.31	0.62	0.58	0.45	0.68	0.64	0.51	0.74	0.47	0.33	0.59			
	Browsing internet	0.43	0.32	0.53	0.50	0.34	0.64	0.37	0.22	0.51	0.49	0.32	0.62	0.40	0.24	0.53			
	Cumulative time	0.66	0.59	0.73	0.68	0.56	0.77	0.66	0.55	0.74	0.75	0.65	0.82	0.60	0.48	0.70			

*ICC* Intraclass Correlation Coefficients, *LCI* Lower Confidence Interval (95%), *UCI* Upper Confidence Interval 336 (95%), *CZ* Czechia, *MX* Mexico

^a^–Small number of responders (n = 9)

**Table 4:** Agreement of dichotomized screen time behaviours using Cohen’s kappa and percentage of consistent responses (no shift)

		All		Boy		Girl		10–12		13–15		16–18	
		Κ	% No Shift	Κ	% No Shift	Κ	% No Shift	Κ	% No Shift	Κ	% No Shift	Κ	% No Shift
All datasets	Gaming	0.65	82.5	0.59	81.2	0.59	83.9	0.65	83.0	0.54	77.0	0.72	87.1
	Social networking	0.64	82.7	0.60	79.8	0.68	85.4	0.71	86.8	0.55	81.4	0.62	81.7
	Watching video	0.47	73.9	0.45	72.4	0.48	75.3	0.55	78.0	0.47	73.4	0.43	72.2
	Browsing internet	0.41	81.6	0.42	82.0	0.40	81.3	0.48	86.2	0.31	76.3	0.49	83.9
	Cumulative time	0.58	85.3	0.52	85.5	0.62	85.2	0.62	84.9	0.47	86.5	0.61	84.5
CZ primary school	Gaming	0.69	84.8	0.61	84.8	0.69	84.8	0.74	90.0	0.65	82.3	0.73^a^	88.9^a^
	Social networking	0.72	86.7	0.69	84.8	0.74	88.6	0.71	86.7	0.67	87.2	0.36^a^	77.8^a^
	Watching video	0.55	78.1	0.61	81.0	0.50	75.2	0.55	76.7	0.55	78.0	0.77^a^	88.9^a^
	Browsing internet	0.35	85.7	0.33	85.7	0.37	85.7	0.40	91.7	0.20	82.3	1.00^a^	100.0^a^
	Cumulative time	0.60	91.0	0.46	86.7	0.77	95.2	0.69	90.0	0.53	90.8	1.00^a^	100.0^a^
CZ high school	Gaming	0.72	87.0	0.65	82.7	0.55	91.7					0.72	87.0
	Social networking	0.62	81.8	0.55	77.8	0.69	86.2					0.62	81.8
	Watching video	0.42	71.7	0.37	68.5	0.43	75.2					0.42	71.7
	Browsing internet	0.45	83.4	0.46	81.5	0.43	85.5					0.45	83.4
	Cumulative time	0.61	84.0	0.63	88.9	0.55	78.6					0.61	84.0
MX primary and secondary school	Gaming	0.49	74.7	0.46	74.8	0.44	74.6	0.58	78.8	0.42	71.4		
	Social networking	0.60	80.3	0.53	77.8	0.61	82.1	0.71	86.9	0.44	75.2		
	Watching video	0.44	73.0	0.39	69.7	0.47	75.4	0.54	78.8	0.36	68.4		
	Browsing internet	0.38	75.6	0.40	78.8	0.36	73.1	0.48	82.8	0.30	69.9		
	Cumulative time	0.51	82.0	0.40	78.8	0.58	84.3	0.58	81.8	0.41	82.0		

Κ - Cohen’s kappa, *CZ* Czechia, *MX* Mexico

^a^- Small number of responders (n = 9)

Time spent watching videos and browsing the internet generally showed moderate reliability. For watching, the ICC was 0.63 (95% CI [0.59, 0.67]), while κ was 0.47 with 73.9% unchanged responses. Browsing the internet yielded the lowest reliability: ICC = 0.52 (95% CI [0.46, 0.57]) and κ = 0.41, with 81.6% unchanged responses.

Subgroup analyses revealed differences across countries and educational levels. Czech primary school students had the highest consistency across most domains, for example, ICCs for gaming and social networking reached 0.76 (95% CI [0.70, 0.81]) and 0.81 (95% CI [0.75, 0.85]), respectively, and κ values for the same domains were 0.69 and 0.72. In contrast, reliability indices were generally lower in the Mexican sample, particularly for browsing and video watching, where ICCs were below 0.43 (95% CI [0.32, 0.53]) and 0.54 (95% CI [0.44, 0.62]) and κ values ranged from 0.38 to 0.44.

Among younger adolescents (10–12 years), ICCs for gaming and social networking were 0.73 (95% CI [0.65, 0.79]) and 0.74 (95% CI [0.66, 0.80]), with κ values of 0.65 and 0.71, respectively. In mid-adolescents (13–15 years), ICCs were lower for browsing (ICC = 0.44, 95% CI [0.34, 0.53]; κ = 0.31). Among older adolescents (16–18 years), where the ICC reached 0.80 (CI [0.76, 0.84]) and κ = 0.72 (87.1%) at gaming. Girls showed slightly better agreement in this domain (κ = 0.68) compared to boys (κ = 0.60), though the ICC values were similar. Gender comparisons suggested slightly higher ICCs for boys in gaming and browsing, whereas girls had higher categorical agreement for social networking.

## Discussion

The primary aim of this study was to assess the test–retest reliability of recreational screen-time items used in the HBSC questionnaire across three diverse adolescent populations within a 2–3-week interval. Overall, the findings indicated that self-reported gaming and social networking could be assessed with moderate to good reliability, whereas video watching and internet browsing were less stable. Reliability also varied by age and sex. Czech students generally demonstrated higher reliability across domains compared to their Mexican counterparts. These results align with earlier studies reporting moderate to good reliability of screen-time measures in Vietnam [37] and China [38]. These findings reinforce the usefulness of the HBSC screen-time items for international youth health monitoring [23, 24]. Specifically, ICCs around 0.50–0.75 are generally sufficient for monitoring group-level patterns, whereas ICCs ≥ 0.90 are typically required for individual-level clinical decisions [40]. By this standard, the moderate-to-good reliability we observed for gaming, social networking and cumulative recreational screen time appears adequate for large-scale surveillance, while lower coefficients for video watching and internet browsing highlight domains that may need further refinement.

Consistent with previous research, the gaming item exhibited the highest test–retest reliability across both continuous (ICC) and categorical (κ) measures, often exceeding ICC values of 0.70. This finding suggested that gaming was a routinized and salient activity that adolescents recalled quite well, likely due to structured and prolonged sessions [42]. Similarly, social networking also demonstrated strong reliability, supported by habitual and frequent usage patterns that facilitate accurate self-reporting [32, 42]. These findings supported the continued inclusion of such items in large-scale adolescent health surveys like HBSC and GSHS.

In contrast, the items on browsing the internet and watching videos demonstrated lower reliability, especially in the Mexican and younger Czech samples. These activities are more fragmented, passive, or multitasked, making them harder to recall accurately. Despite relatively high percentages of “no shift” responses for browsing the internet or seeking information online, the ICC and κ values were lower, indicating reduced stability in time estimation [31, 43]. This is consistent with studies showing that over 79% of youth underestimate daily smartphone use compared to objective tracking data [43].

Age emerged as a significant factor affecting response consistency. Older adolescents (16–18 years) consistently reported higher test–retest agreement, especially for gaming and social networking. This aligns with cognitive development research suggesting improved memory and behavioral stability at this stage [10, 26, 38]. Middle adolescents (13–15 years) showed the lowest reliability, particularly for browsing and video watching, likely due to greater variability in use and experimentation with digital content. Younger adolescents (10–12 years) showed stronger reliability in some domains, though results varied by activity. At this age, lower overall screen exposure may reflect greater parental control and clearer household rules regarding media use [44], as well as cross-cultural differences. A Vietnamese study similarly reported that social media use was less stable in younger teens [37].

Gender-based differences also followed expected patterns. Boys demonstrated higher reliability for gaming and browsing, while girls reported more consistent responses for social networking. These results mirror established gender-based differences in digital preferences and behaviour [18, 45, 46]. These patterns suggest that gender-sensitive adaptations in item design may improve measurement precision.

Cross-national comparisons revealed important contrasts. Czech students, particularly from primary schools, exhibited higher reliability across screen-based behaviours compared to their Mexican peers. These differences may stem from cultural norms, variations in digital literacy, or access to technology. Methodological aspects may also have contributed. In the Czech Republic, surveys were administered by research staff without teacher supervision to reduce bias. In Mexico, surveys were likewise conducted without teacher supervision; however, some students reported difficulties in understanding certain items and occasionally required clarification from data collectors. Such challenges, together with evidence that adolescents may struggle to accurately differentiate between types of screen use or may inadvertently respond to the wrong item (as also observed in national surveys), could partly explain the lower reliability observed among Mexican participants [35, 37].

Methodologically, the use of both continuous (ICC) and categorical (κ) reliability indicators enabled a comprehensive assessment of temporal consistency. The moderate-to-good reliability of gaming and social networking items supported their continued use in adolescent surveillance. However, domains such as browsing and video watching may require refinement. Prior research has found that such incidental behaviours are more difficult to estimate accurately [31, 32]. Future iterations of the questionnaire might benefit from improved behavioural framing, time-estimation aids, or integration of objective measures such as screen-logging apps.

This study has several strengths and limitations. One of its key strengths was that, to our knowledge, it was the first study to examine the reliability of screen-time items included in the current HBSC study protocol and applied across multiple countries. Furthermore, the study attempted to test this questionnaire across different countries and cultural contexts with a high number of participants, which significantly enhanced its relevance and importance. However, several limitations must be acknowledged. First, the test–retest interval varied slightly (2–3 weeks). This time window was chosen as a methodological compromise that reduces the probability that adolescents simply remember their initial answers while remaining short enough to avoid major developmental or seasonal changes in screen-time habits. We therefore consider it unlikely that this modest variation materially biased the reliability estimates, although some influence cannot be completely ruled out. Second, the screen-time items were originally measured on an ordinal response scale but were treated as continuous for ICC analyses, which assumes interval-level measurement; therefore, the resulting ICC values should be interpreted with caution. In addition, some age-by-country subgroups had very small cell sizes, leading to wide confidence intervals for the ICCs in these subgroups. Third, changes in responses may reflect true behavioural shifts, particularly among younger adolescents. Fourth, the Mexican cohort was limited to private school students, reducing national representativeness. Thus, children from low-socioeconomic backgrounds may be underrepresented in our sample. Finally, the Czech high school sample only included students aged 16–18y, limiting direct cross-country comparisons across the full adolescent age range.

Although our results did not exceed the threshold for “excellent” reliability (ICC ≥ 0.9), they strengthened the case for using HBSC screen-time indicators—particularly for gaming and social networking—in international adolescent health research and surveillance. These items appeared to capture habitual digital behaviours with acceptable consistency, comparable to other health-related self-report measures [35]. However, less structured behaviours such as internet browsing or seeking information online and video watching showed weaker reliability, highlighting the need for methodological refinement.

As digital habits continue to evolve—with increases in streaming, background media use, and multitasking—there is a growing need for tools that better capture the content, context, and purpose of screen time [31, 32]. Future efforts should prioritize the development of culturally sensitive, age-appropriate, and gender-responsive measures that balance self-report practicality with validation from objective tracking sources. Nevertheless, in contexts where short and feasible instruments are needed, the HBSC screen-time items—particularly those addressing gaming and social networking—can be recommended as reliable and practical measures for adolescent health monitoring.

## Data Availability

The datasets generated and/or analysed during the current study are available from the corresponding author on reasonable request.
